# A functional inflammasome activation assaydifferentiates patients with pathogenic NLRP3mutations and symptomatic patients with lowpenetrance variants

**DOI:** 10.1186/1546-0096-13-S1-O74

**Published:** 2015-09-28

**Authors:** N Rieber, A Gavrilov, L Hofer, A Singh, H Öz, T Endres, I Schäfer, R Handgretinger, D Hartl, J Kümmerle-Deschner

**Affiliations:** 1 Children's hospital Tübingen, Tübingen, Germany

## Question

The cryopyrin-associated periodic syndromes (CAPS) are characterized by recurrent episodes of systemic inflammation. CAPS is caused by mutations in the *NLRP3* gene encoding cryopyrin, an important component of the NLRP3 inflammasome that activates caspase-1 resulting in inflammation by excessive production of IL-1β and others. Besides confirmed pathogenic NLRP3 mutations, patients with CAPS-like symptoms frequently show low penetrance variants in NLRP3. The disease relevance of these variants is inconsistent. The analysis of IL-1β in the serum did not prove to be a valid diagnostic test in these individuals.

## Methods

In this study, we investigated if an inflammasome activation assay differentiates between patients with confirmed pathogenic CAPS mutations, patients with low penetrance NLRP3 variants (V198M and Q703K) and healthy controls. The study population consisted of 17 patients with genetically proven Muckle-Wells syndrome, 11 patients with low penetrance *NLRP3* variants and 15 healthy controls. Concentrations of IL-1β, IL-18, Caspase-1, TNF-α and IL-6 were quantified in cell culture supernatants after inflammasome stimulation with LPS and LPS + ATP for several time intervals.

## Results

The release of mature IL-1β, IL-18, and caspase-1 into cell culture supernatants after 4h of inflammasome stimulation was significantly increased in patients with confirmed pathogenic CAPS mutations compared to low penetrance NLRP3 variants and controls. IL-1β secretion in CAPS patients correlated with disease severity. TNF-α secretion was significantly reduced in CAPS patients and NLRP3 variants when compared to healthy controls after 4h of stimulation.

## Conclusion

This inflammasome activation assay differentiates between autoinflammation patients with confirmed pathogenic CAPS mutations and patients with low penetrance NLRP3 variants, and points towards alternative pathophysiological mechanisms in low penetrance NLRP3 variants.

